# Identification of the Plasmid-Mediated Colistin Resistance Gene *mcr*-1 in *Escherichia coli* Isolates From Migratory Birds in Guangdong, China

**DOI:** 10.3389/fmicb.2021.755233

**Published:** 2021-10-21

**Authors:** Yan Zhang, Xu Kuang, Juan Liu, Ruan-Yang Sun, Xing-Ping Li, Jian Sun, Xiao-Ping Liao, Ya-Hong Liu, Yang Yu

**Affiliations:** ^1^Guangdong Provincial Key Laboratory of Veterinary Pharmaceutics Development and Safety Evaluation, South China Agricultural University, Guangzhou, China; ^2^College of Animal Science and Technology, Henan University of Science and Technology, Luoyang, China; ^3^Guangdong Provincial Key Laboratory of Microbial Safety and Health, Guangdong Institute of Microbiology, Guangdong Academy of Sciences, Guangzhou, China; ^4^Guangdong Laboratory for Lingnan Modern Agriculture, Guangzhou, China

**Keywords:** migratory birds, *mcr-1*, *Escherichia coli*, transmission, antibiotic resistance

## Abstract

We determined the prevalence and transmission characteristics of *mcr-1*-positive *Escherichia coli* (MCRPEC) isolates from migratory birds *Anser indicus* in Guangdong, China. We identified 22 MCRPEC from 303 *A. indicus* fecal samples (7.3%) in Guangzhou, Zhaoqing, and Futian. The *mcr-1* gene coexisted with 24 other types of antibiotic resistance genes (ARG), and 11 ARGs were highly prevalent at levels >50%. The MCRPEC displayed a diversity of sequence types (ST), and 19 distinct STs were identified with ST10, ST1146, and ST1147 as the most prevalent. In addition, these MCRPEC from birds were closely related phylogenetically to those from other sources in China. Whole-genome sequencing analysis demonstrated that *mcr*-1 was located on IncX4 (*n*=9, 40.9%), IncI2 (*n*=5, 22.7%) and IncP (*n*=1, 4.5%) plasmids and the latter shared an identical plasmid backbone with other sources. These results highlight the significance of migratory birds in the transmission of antibiotic resistance and provide powerful evidence that migratory birds are potential transmitters of antibiotic resistance.

## Introduction

Colistin is a polymyxin antibiotic that has been used in veterinary clinics for many years. It is now applied as a last-resort drug for treatment of carbapenem-resistant Enterobacterial infections (Mendes et al., 2018). The emergence of the plasmid-mediated mobile colistin resistance gene *mcr-1* in China and its rapid global spread now poses a serious threat to public health (Liu et al., 2016; Wang et al., 2018). Significantly, these *mcr-1* plasmids often harbor other antibiotic resistance genes (ARG) that encode carbapenemase and extended-spectrum β-lactamase (ESBL) resistance (Haenni et al., 2016; Yao et al., 2016). Therefore, the global distribution and spread of *mcr-1* gene is concerning.

The World Health Organization (WHO) has developed a global strategy to address antimicrobial resistance based on the “One Health” approach that is based on the close links between human, animal, and environmental health (Dafale et al., 2020). Wildlife can act as ARG reservoirs and transmission vectors between microorganisms and the environment (Allen et al., 2010; Arnold et al., 2016). Therefore, a contaminated environment also plays a key role in the spread of antibiotic resistance since this is where bacteria from different sources are able to exchange ARGs (Aeksiri et al., 2019). In particular, birds play a large part in ARG dissemination due to their environmental exposure through ingested food or polluted water (Jarma et al., 2021). This is especially true for migratory birds that can act as ARG reservoirs and spread antibiotic resistance through migration (Vergara et al., 2017; Elsohaby et al., 2021). For instance, the *mcr*-1 gene has been isolated from European herring gulls (Ruzauskas and Vaskeviciute, 2016), from Spanish and Portuguese gulls (Ahlstrom et al., 2021) as well as in Thailand and China in migratory birds (Aeksiri et al., 2019; Lake, 2020).

Since colistin is considered the last-resort antibiotic used to treat multidrug-resistant bacteria (Li et al., 2020), the emergence of the *mcr-1* gene in migratory birds is especially worrisome. Monitoring the level of antibiotic resistance carried in migratory birds is a necessary step to prevent ARG spread. This study investigated the prevalence and genomic structures of *mcr-1* producing *Escherichia coli* (MCRPEC) isolated from migratory birds *Anser indicus* in Guangdong, China.

## Materials and Methods

### Ethics Statement

The Institutional Review Board of South China Agricultural University (SCAU-IRB) approved the Samples and bacteria protocols. All *A. indicus* feces were sampled under authorization from Animal Research Committees of South China Agricultural University (SCAU-IACUC).

### Sampling Information

A total of 303 feces samples from *A. indicus* were collected in 2017 in Guangzhou, Zhaoqing, and Futian in Guangdong province. Briefly, all samples were placed to 1.0ml of LB Broth and incubated for 16–18h at 37°C followed by inoculation on MacConkey agar plates containing 2.0mg/L colistin that were incubated for 16h. Two or three different forms red colonies were selected for identification using the MALDI-TOF 80 MS Axima system (Shimadzu-Biotech Corp., Kyoto, Japan) and 16S rRNA sequencing. The colistin-resistant isolates were screened for the presence of the *mcr*-1 gene by PCR as previously described (Yang et al., 2019a).

### Antimicrobial Susceptibility Testing

The antimicrobial sensitivity of 22 MCRPE for 15 antibiotics was examined by measuring the minimal inhibitory concentration (MIC) using the agar dilution method and interpreted according to the Clinical and Laboratory Standards Institute guidelines (CLSI M100-S28). Susceptibility to colistin was performed by broth micro-dilution as recommended by the European Committee on Antimicrobial Susceptibility Testing (EUCAST 92 Version 6.0; EUCAST, 2016). Quality control of the procedure was conducted by using the susceptible *E. coli* ATCC 25922.

### Whole-Genome Sequencing and Bioinformatics Analysis

Total genomic DNA was extracted from 22 MCRPEC isolates using a Genomic DNA Purification Kit (Tiangen, Beijing, China) as per the manufacturer’s instructions. WGS was performed with the Illumina Hiseq 2500 System (Novogene Guangzhou, China) using the paired-end 2×150-bp sequencing protocol. Raw sequence reads were trimmed using Trim Galore, and the genomes were *de novo*-assembled into contigs using SPAdes (3.9.0) predefined kmers set. All genome assemblies of 22 *E. coli* isolates were deposited in GenBank and are registered with BioProject number PRJNA748548.

### Bioinformatics Analysis

The CGE platform^1^ was used for analyses of multilocus sequence typing (MLST-2.0), acquired resistance genes (ResFinder 4.1, all antibiotic resistance databases were selected with a cut-off value of 95% identity and 80% minimum coverage) and plasmid incompatibility groups (PlasmidFinder-2.1 version, using Enterobacteriaceae database with parameters of minimum 95% identity and 85% query coverage). Sequence comparisons between *mcr*-1 carrying plasmids were performed using Easyfig and Brig (Alikhan et al., 2011). Phylogenetic trees for MCRPEC isolates in this study were constructed using homologous strains from the NCBI database using CSI Phylogeny (v1.4), and *E. coli* (U4) was used as the reference genome (Kaas et al., 2014). The corresponding characteristics of each isolate visualized using FigTree v1.4.4 and the online tool iTOL.^2^ Single polynucleotide pairs (SNP) analysis was visualized as a heatmap of the SNP count matrix using BacWGSTdb 2.0 (Feng et al., 2021). The sequences of *mcr*-1 plasmids were constructed by *de novo* assembly using Genious (10.0.7). Circular comparisons among *mcr*-1-related IncX4 and IncI2 plasmids from this study and NCBI database were performed using BLAST Ring Image Generator (BRIG v0.9555).

## Results

### Bacterial Isolation and Antibiotic Susceptibility Testing

We recovered 22 (7.3%) MCRPE from 303 *A. indicus* fecal samples in Guangdong Province, China, that included 10% from Futian (12/120), 5.7% from Zhaoqing (2/35) and 5.4% from Guangzhou (8/148). The MIC values for colistin for these isolates were all ≥4μg/ml. These isolates also possessed high levels of resistance to other common antibiotics including sulfamethoxazole/trimethoprim (95.4%), fosfomycin (81.8%), florfenicol (81.8%), tetracycline (77.2%), ciprofloxacin (34.1%), and olaquindox (34.1%). Smaller numbers of isolates displayed resistance to nalidixic acid (27.3%), cefotaxime (22.7%), streptomycin (18.1%), cefoxitin (13.6%), gentamicin (4.5%), and amikacin (4.5%). Notably, all strains remained sensitive to meropenem and tigecycline (Table 1; Supplementary Figure S2).

**Table 1:** Bacterial information and antimicrobial resistance profiles.

Isolate	Plasmid type	Resistance phenotype
FT104-1	IncI2	CS-FOS-OLA-FFC-TET-S/T
FT105-1	IncI2	CS-FOS-OLA-GEN-AMK-FOX-FFC-TET-S/T
FT109-1	IncP	CS-FOS-STR-OLA-NAL-CIP-CTX-FFC-TET-S/T
FT11	Untypable	CS-FOS-OLA-CIP-FFC-TET-S/T
FT130-1	IncX4	CS-FOS-FFC-TET-S/T
FT130-2	Untypable	CS-STR-OLA-CIP-FFC-TET-S/T
FT13-1	IncX4	CS-FOS-STR-OLA-CIP-FFC-TET-S/T
FT18-1	IncX4	CS-FOS-OLA-CIP-FFC-S/T
FT30-1	IncX4	CS-FOS-FFC-TET
FT7-2	IncX4	CS-FOS-OLA-CIP-FFC-S/T
FT95-1	Untypable	CS-NAL-CIP-CTX-FFC-TET-S/T
FT99	IncI2	CS-FOS-OLA-CIP-FFC-TET-S/T
U23	IncI2	CS-FOS-TET-S/T
U34-1	IncX4	CS-FOS-STR-S/T
U39-1	IncX4	CS-FOS-NAL-CIP-CTX-FFC-TET-S/T
U4	IncI2	CS-FOS-TET-S/T
U40	Untypable	CS-STR-BAL-CIP-CTX-FOX-FFC-TET-S/T
U48-1	IncX4	CS-FOS-FFC-TET-S/T
U48-2	Untypable	CS-FOS-OLA-FFC-TET-S/T
U7	Untypable	CS-S/T
ZQ28	IncX4	CS-FOS-OLA-NAL-CTX-FFC-TET-S/T
ZQS2-1	Untypable	CS-FOS-FOX-FFC-TET-S/T

CS, colistin; FOS, Fosfomycin; STR, streptomycin; OLA, olaquindox; NAL, nalidixic acid; GEN, gentamicin; AMK, amikacin; FOX, cefoxitin; CTX, cefotaxime; FFC, florfenicol; CIP, ciprofloxacin; TET, tetracycline; S/T, trimethoprim/sulfamethoxazole.

### Whole-Genome Sequencing Analysis

**Figure 1:**
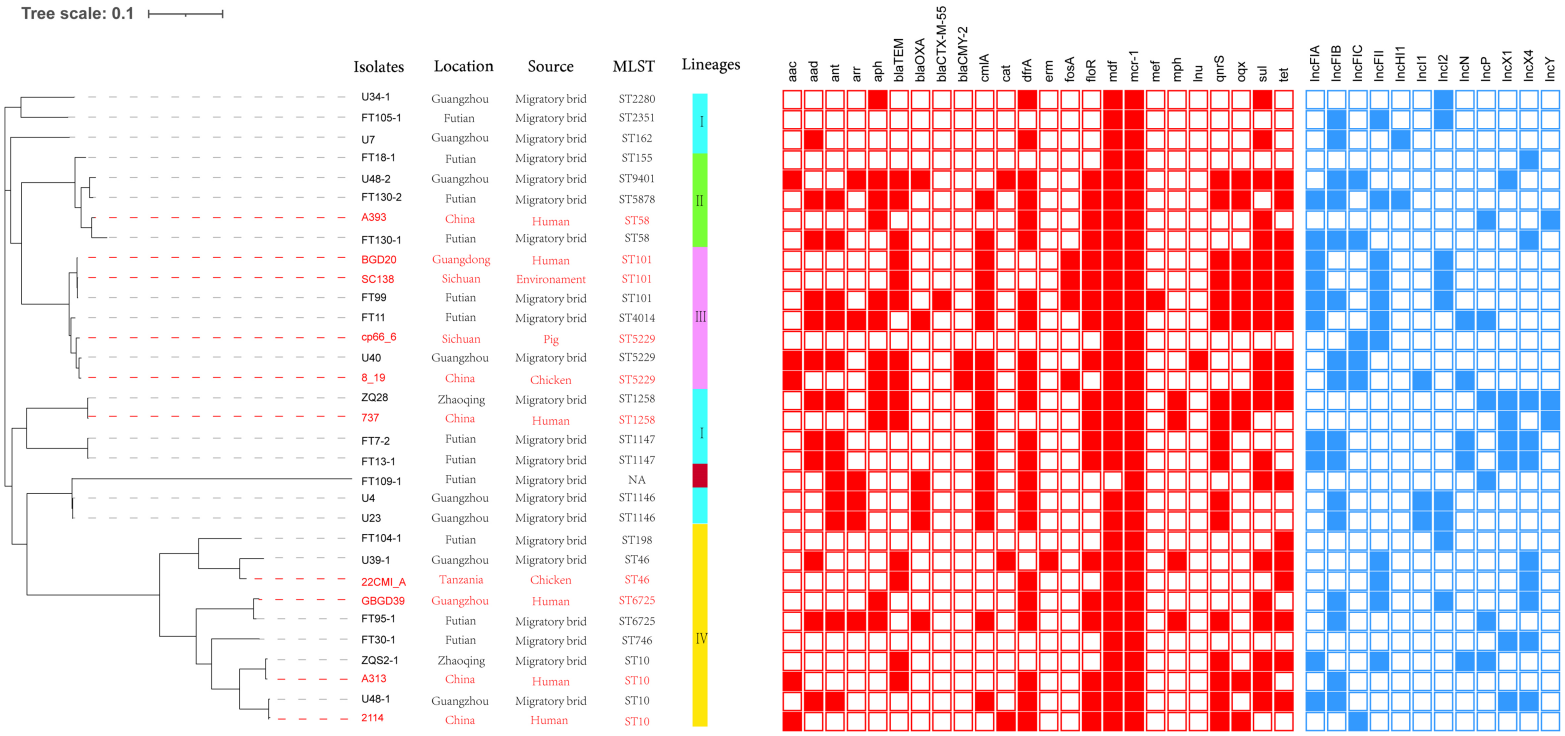
Analysis of MCRPE isolates used in this study. **Left Panel:** Isolates from migratory birds in Guangdong, China (black font) and from the NCBI database (red font). **Right Panel:** Genetic relationships among 32 *mcr-1* positive *Escherichia coli* (MCRPEC) isolates using a maximum likelihood tree. Red-filled squares indicate possession of the indicated antibiotic resistance genes (ARG) and blue-filled squares indicated plasmid Inc type.

We sequenced 22 of MCRPE isolates using the Illumina HiSeq platform, and the average genome size was 4.9Mbp. Interestingly, we identified 19 distinct STs that included ST10 (2/22), ST1146 (2/22), and ST1147 (2/22) and the remaining were single STs that included ST198, ST2351, ST4014, ST58, ST5878, ST155, ST746, ST6725, ST101, ST2280, ST46, ST5229, ST9401, ST162, and ST1258. The strain FT109-1 was a member of a previously unreported new ST (Figure 1).

We reconstructed a phylogenetic tree using the 22 MCRPE isolates along with 10 MCRPE isolates from the NCBI database. All isolates were classified into four clades lineage except FT109-1 (see above). The major lineage IV included 10 (31%) isolates, and ST10 members accounted for 40% of this lineage. In addition, we also found close phylogenetic relationships between our 22 MCRPE from *A. indicus* and other sources in China that included humans, pigs, chickens, and environmental samples.

To further assess the relationships between these isolates, we performed a SNP analysis that indicated most of our MCRPE isolates had significant SNP differences. Notably, in two cases, there were two isolates possessing a collection of different genomic characteristics that were recovered from the same sample (FT130 and U48; Table 1). For instance, both ST10 (U48-1) and ST9401 (U48-2) were recovered from sample 18FS1 and shared 42,580 SNPs (Supplementary Figure S1).

Additionally, our MCRPE group represented 24 ARG types that conferred resistance to 14 classes of antibiotics including colistin, fosfomycin, streptomycin, nalidixic acid, ciprofloxacin, florfenicol, cefotaxime, cefoxitin, tetracyclines, gentamicin, amikacin, olaquindox, and sulfamethoxazole/trimethoprim. Among these, 11 ARGs were highly prevalent with detection rates >50% and included *aad*, *aph*, *cmlA*, *dfrA*, *floR*, *mdf*, *mcr-1*, *qnrs*, *oqx*, *sul*, and *tet* (Table 1; Figure 1).

### *mcr*-1-Associated Plasmid Types

**Figure 2:**
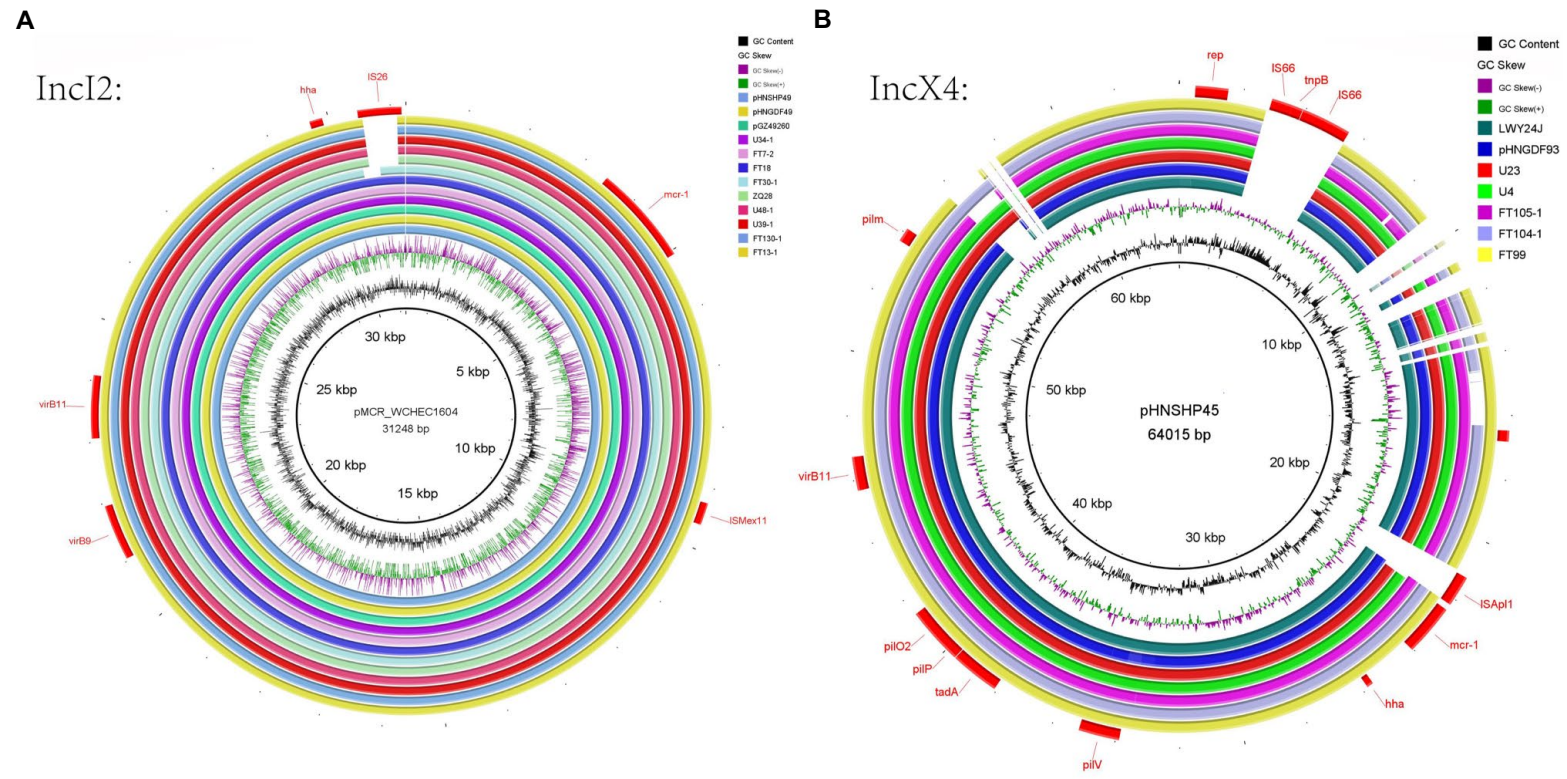
IncI2 plasmid analysis for 21 MCRPEC isolates from migratory birds **(A)**. Alignments of IncI2 plasmids from this study with reference plasmid pHNSHP45. **(B)** Alignment of IncX4 plasmids with reference plasmid pMCR_WCHEC1604. The sources of these plasmids were migratory birds (FT7-2, FT18, FT13-1, FT30-1, FT99, FT104-1, FT105-1, FT130-1, U4, U23, U34-1, U39-1, U48-1, and ZQ28), human plasmids (pMCR_WCHEC1604, pHNSHP45, and pGZ49260) and animals (pNFGDF93, pLWY24J, pNFGDF49, and pHNSHP49).

Whole-genome sequencing analysis indicated that 12 different Inc types were present on plasmids in the 22 MCRPEC isolates (Figure 1). In 15/22 isolates, we could identify the replicon sequence type of *mcr*-1-harboring plasmids and included IncX4 (*n*=9, 40.9%), IncI2 (*n*=5, 22.7%) and IncP (*n*=1, 4.5%). Alignment of *mcr*-1-carrying IncX4 and IncI2 plasmids demonstrated that all *mcr*-1-carrying plasmids shared identical plasmid backbones. More importantly, the backbones of these mcr-1-linked plasmids from *A. indicus* were highly similar to those recovered from other sources including diverse animals, humans, and poultry. Plasmids used for comparison include the following: human plasmids: pMCR_WCHEC1604 (KY829117), pHNSHP45 (KP347127.1), pGZ49260 (MG210937.1) and animals: pNFGDF93 (MF978388.1), pLWY24J (MN689940.1), pNFGDF49 (MF978387.1), and pHNSHP49 (MF774188.1). Interestingly, the insertion element *ISApl1* was not present in our *mcr-1*-associated IncI2 plasmids and *IS26* was ether partially or totally absent from the mcr-1-carrying IncX4 plasmids (Figure 2).

**Figure 3:**
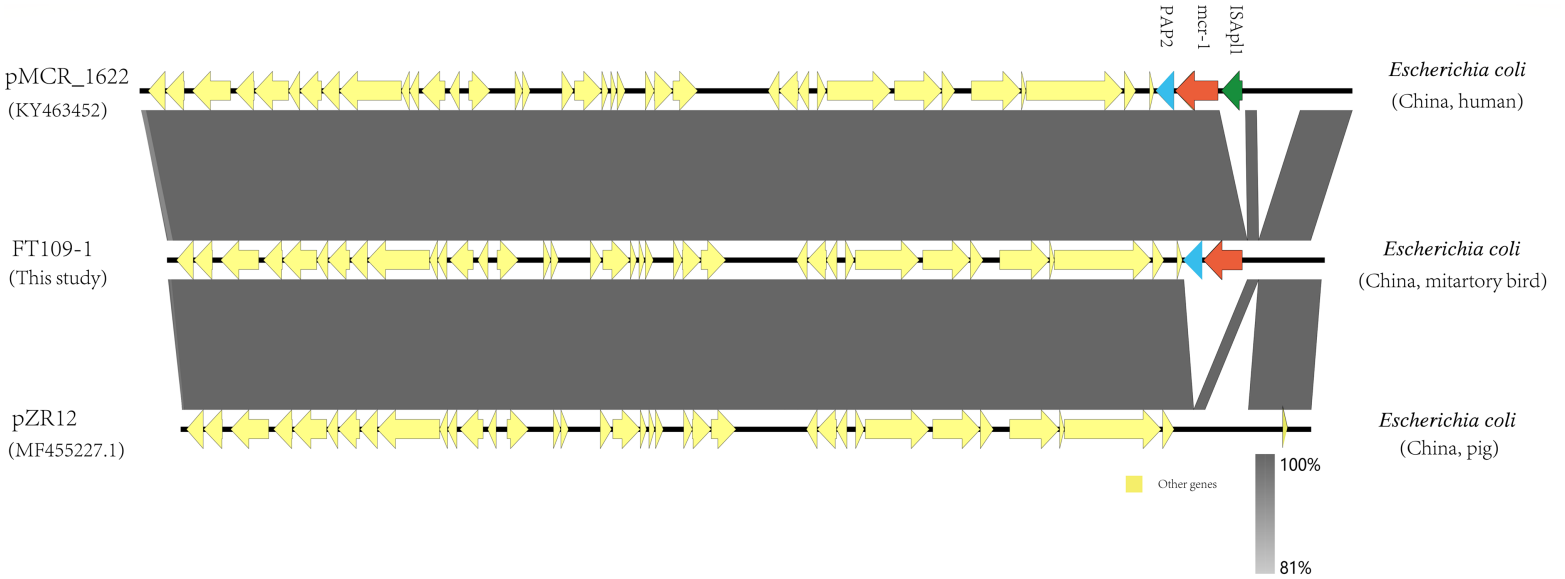
Genomic organization between pFT109-1 and the indicated plasmids obtained from GenBank.

Additionally, we also found the presence of broad-host-range IncP plasmids that acted as *mcr*-1 vectors in this study. The *mcr*-1-carrying IncP plasmids shared conserved backbones with the plasmids pMCR_1622 and pZR12 taken from *E.coli* human and pig isolates, respectively (Figure 3). *Mcr-1-PAP2* and *ISApl1-mcr-1-PAP2* were inserted within the same locus in pMCR_1622 and pZR12 and suggested that this locus is a hot spot for the insertion of *ISApl1* (see above).

## Discussion

The plasmid-mediated colistin resistance gene *mcr-1* was originally identified in *E. coli* and has since been found in many countries on all continents (Liu et al., 2016; Matamoros et al., 2017). The speed and extent of the spread of the *mcr-1* gene are worrying especially since migratory birds carry foreign ARGs and act as a major source of bacterial antibiotic resistance in local environments (Lake, 2020). Long-distance migratory birds are considered key agents in the spread of antibiotic resistance (Franklin et al., 2020). For antibiotic-resistant bacteria, aquatic systems are major traffic routes between wildlife and humans (Taylor et al., 2011). The resistance genes acquired by birds can be re-introduced in the environment, possibly into different water systems (e.g., by migrating ducks) and might re-infect humans directly *via* contact with contaminated water or indirectly by the introduction of these genes into the food chain (Marcelino et al., 2019), and long-distance migration of migratory birds may accelerate this spread, which poses a great threat to human safety (Chen et al., 2017; Hadjadj et al., 2017; Monte et al., 2017; Mohsin et al., 2019). Therefore, it is very necessary to monitor the flora of migratory birds. In our study, we isolated 22 MCRPEC isolates in fecal samples from *A. indicus* in Guangdong, China. This is the first report of the presence of MCRPEC from migratory birds in Guangdong, and our study provides strong evidence that migratory birds are potential transmitters of the colistin resistance gene *mcr-1*.

Whole-genome sequencing analysis of these *mcr-1* positive *E. coli* strains identified 19 different STs and was consistent with previous reports documenting the ST diversity of MCRPEC (Wang et al., 2018). However, ST10, ST1146, and ST1147 were more prevalent in our isolates and ST10 is frequently found in clinical (Lin et al., 2020), animal (García-Meniño et al., 2018), and environmental samples (Flament-Simon et al., 2020). In addition, we found that the genomes of two MCRPE isolates from the same sample differed by the phylogenetic analysis. This scenario is worrying and indicated that a significant diversity was present in our population of MCRPE isolates from migratory birds. More notably, the emergence and spread of *mcr-1* gene in humans, animals, and the environment have been reported globally. In our study, the close phylogenetic relationships for 22 of the MCRPEC isolates from *A. indicus* and other sources in China indicated the existence of clonal transmission of *mcr-1* in migratory bird populations, and the latter are likely responsible for the widespread occurrence of *mcr-1*. This indicated that migratory birds played an important role in the transmission of the *mcr*-1 gene.

In addition to *mcr-1*, we also identified numerous ARGs in MCRPEC isolates such as the plasmid-mediated quinolone resistance gene *qnrS1*, the tetracycline resistance gene *tet,* and ESBL-dependent *bla*_CTX-M-55_ genes. Many previous studies have reported high levels of ARGs in the intestinal flora of migratory birds (Jarma et al., 2021) and thereby indicated that antibiotic resistance can easily be disseminated *via* migration (Elsohaby et al., 2021). According to a survey, *mcr-1* resistance gene is widespread among different birds, accounting for 50% of the total samples (Cao et al., 2020). Notably, silent transmission of *mcr-1* gene was observed, and MCRPE may be difficult to detect whether the *mcr-1* gene is only tested for in colistin-resistant isolates (Moura et al., 2016). As the reservoir of resistance genes, the resistance level of the strains carried by migratory birds should continue to be concerned.

Whole-genome sequencing analysis of our isolates further revealed the presence of different *mcr-1* harboring plasmids, and the IncX4 and IncI2 types were the most prevalent. The IncX4 and IncI2 type plasmids have a high transferability, and this creates favorable conditions for horizontal transmission of *mcr-1* (Yang et al., 2019b). IncX4-type plasmids represent promiscuous plasmids contributing to the intercontinental spread of the *mcr-1* gene (Fernandes et al., 2016), which obtained from different bacterial species, belonging to different STs, isolated in different clinical contexts, including wildlife, and found on different continents are highly similar in the plasmid backbone sequences. The ubiquity and transferability of IncX4 plasmids carrying *mcr-1* sheds light on the role of this incompatibility group in the global spread of colistin resistance (Sellera et al., 2017). In our study, nine IncX4 plasmids and five IncI2 plasmids carrying *mcr-1* in our study shared the same plasmid skeletons as isolates acquired from humans, animals, and the environment as reported elsewhere (Figure 3). This indicated that these plasmid types have been diffusing between humans, animals, and the environment (Matamoros et al., 2017), IncX4 and IncI2 plasmids facilitated the transmission of the *mcr*-1 gene. The additional presence of an *mcr-1-pap2* cassette (Yang et al., 2017) can also function as an independent mobile genetic that could facilitate ARG acquisition and mobilization between bacteria by a mechanism known as “copy-paste and paste” (Partridge et al., 2018). For instance, one or two copies of *ISApl1* that flank the *mcr*-1-*pap*2 cassette can actively capture and mobilize *mcr*-1 genes.

Together our data provide evidence for the horizontal transfer of *mcr-1* due to migratory bird long-distance migrations. *ISApl1* is a common mobile genetic element found in *mcr*-1 plasmids (Snesrud et al., 2016). Interestingly, IS*Apl1* and other mobile elements were not present in the *mcr-1*-related IncX4 and IncI2 plasmid backgrounds in our study isolates. The reason for this was most likely that IS*Apl1* was lost during subsequent recombination steps following the initial mobilization of *mcr*-1 (Snesrud et al., 2016). In addition, in this study, *mcr-1* was also carried by the broad-host-range IncP type plasmid and shared conserved backbones with *E. coli* plasmids pMCR_1622 and pZR12 from human and pig samples, respectively. Moreover, IncP plasmids have been reported to mediate the transmission of *mcr-1* across various hosts and has the potential to become a dominant *mcr*-1 carrier (Zhao et al., 2017; Fish, 2018). The emergence of a large group of host plasmids carrying *mcr-1* in long-haul migratory birds is of great concern for public health and further suggests that the drug-resistant flora in migratory birds should be monitored.

## Conclusion

In conclusion, we identified 22 *mcr-1*-positive *E. coli* isolates from migratory birds *A. indicus* in Guangdong, China. Notably, this is the first study to report the development of diversity in the population of MCRPE isolates from migratory birds in Guangdong. Phylogenetic analysis proved that the development of diversity in the population of MCRPE isolates from migratory birds. WGS analysis further determined that *mcr-1* coexisted with other ARGs and also demonstrated diversity in the plasmid population, and the latter provides important epidemiological information for the global dissemination of the *mcr-1* gene. These plasmids can serve as vectors for rapid spread of colistin resistance among different hosts over long distances due to bird migration. The antibiotic-resistant flora in migratory birds should therefore be under constant surveillance.

## Data Availability Statement

The datasets presented in this study can be found in online repositories. The names of the repository/repositories and accession number(s) can be found at: https://www.ncbi.nlm.nih.gov/, PRJNA748548.

## Author Contributions

YZ and X-PLi: sampling. YZ and R-YS: data analysis. YZ: conceptualization, methodology, and writing-first draft. XK and JL: investigation and writing – review and editing. YY, JS, X-PLa, and Y-HL: conceptualization, writing – review and editing, supervision, and funding acquisition. All authors contributed to the article and approved the submitted version.

## Funding

This study was jointly supported by the National Natural Science Foundation of China (31730097); the Local Innovative and Research Teams Project of Guangdong Pearl River Talents Program (2019BT02N054); the Program for Changjiang Scholars and Innovative Research Team in University of Ministry of Education of China (Grant No. IRT13063); and the innovation Team Project of Guangdong University (2019KCXTD001).

## Conflict of Interest

The authors declare that the research was conducted in the absence of any commercial or financial relationships that could be construed as a potential conflict of interest.

## Publisher’s Note

All claims expressed in this article are solely those of the authors and do not necessarily represent those of their affiliated organizations, or those of the publisher, the editors and the reviewers. Any product that may be evaluated in this article, or claim that may be made by its manufacturer, is not guaranteed or endorsed by the publisher.

## Footnotes

^1^ http://www.genomicepidemiology.org/

^2^ https://itol.embl.de/

## Supplementary Material

Supplementary Figure S1 Single polynucleotide pair (SNP)-heatmap analysis of MCRPE isolates used in this study using SNP count matrices.

Supplementary Figure S2 The antimicrobial sensitivity of 22 MCRPE for 15 antibiotics, yellow indicates that the strain is not sensitive to the drug (CS, colistin; FOS, Fosfomycin; STR, streptomycin; OLA, olaquindox; NAL, nalidixic acid; GEN, gentamicin; AMK, amikacin; FOX, cefoxitin; CTX, cefotaxime; FFC, florfenicol; CIP, ciprofloxacin; TET, tetracycline; MEM, meropenemS/T; TIG, tigecycline; trimethoprim/sulfamethoxazole).
