# Supplementary figures and images for: Identification of the Plasmid-Mediated Colistin Resistance Gene mcr-1 in Escherichia coli Isolates From Migratory Birds in Guangdong, China
Source: Front Microbiol. 2021 Oct 21;12:755233. doi: 10.3389/fmicb.2021.755233 (PMC8567052; doi:10.3389/fmicb.2021.755233)

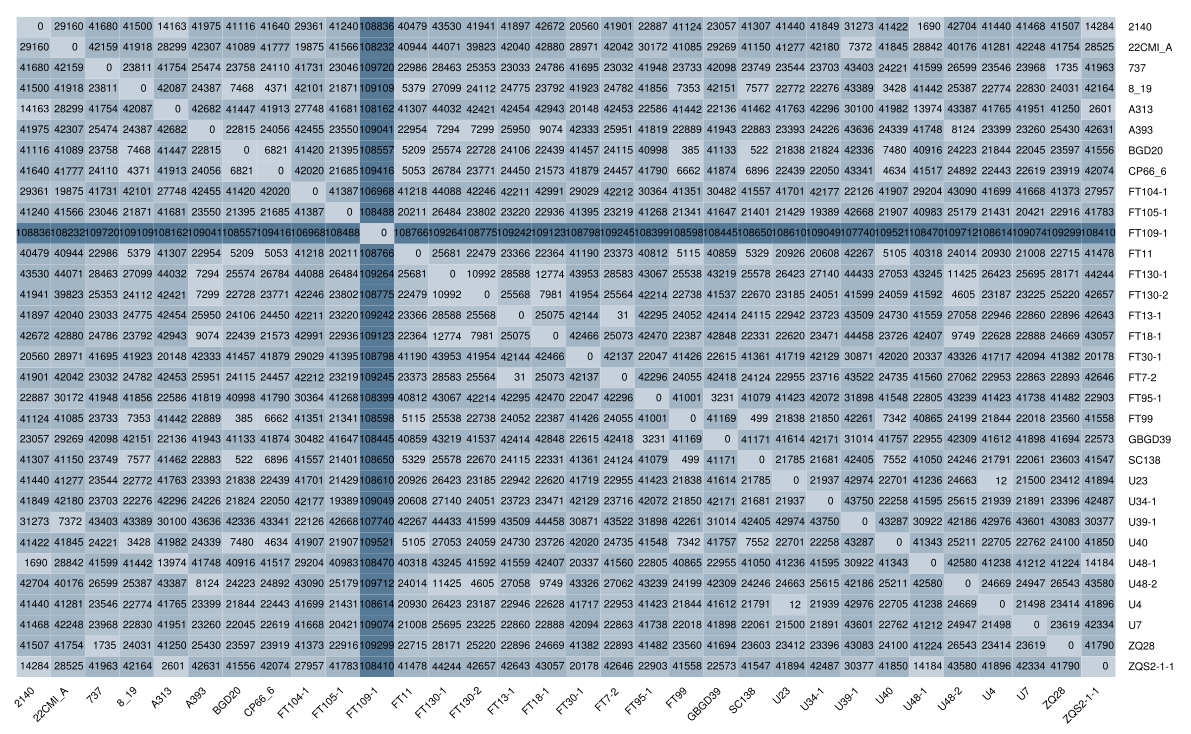

Supplement: Supplementary Figure S1 — Single polynucleotide pair (SNP)-heatmap analysis of MCRPE isolates used in this study using SNP count matrices. [file Image_1.TIF]

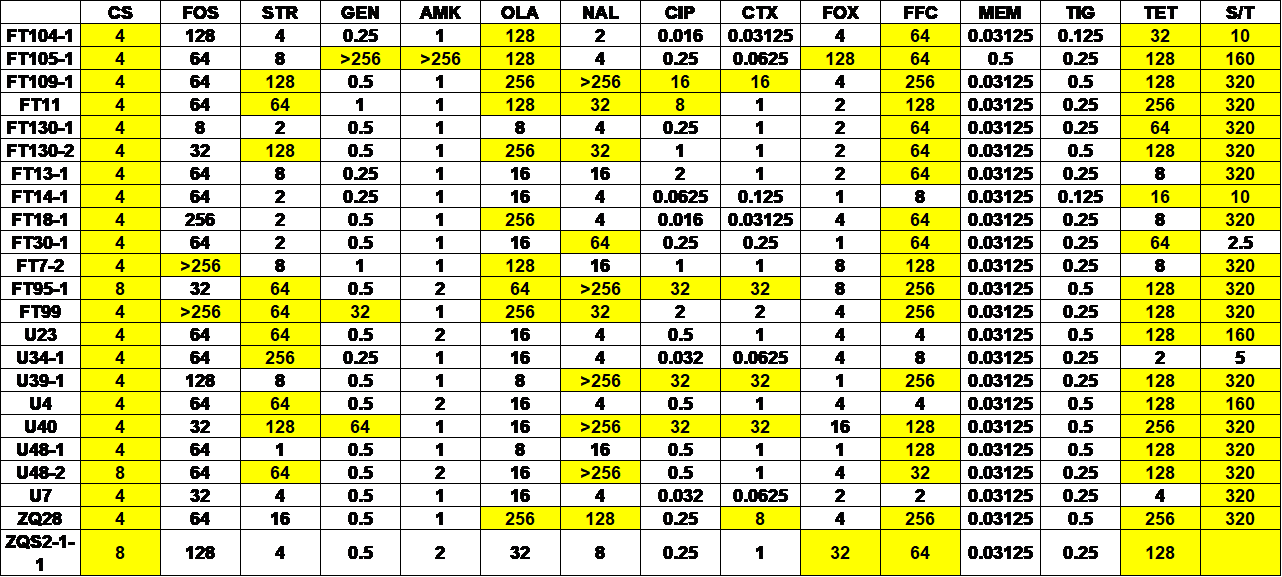

Supplement: Supplementary Figure S2 — The antimicrobial sensitivity of 22 MCRPE for 15 antibiotics, yellow indicates that the strain is not sensitive to the drug (CS, colistin; FOS, Fosfomycin; STR, streptomycin; OLA, olaquindox; NAL, nalidixic acid; GEN, gentamicin; AMK, amikacin; FOX, cefoxitin; CTX, cefotaxime; FFC, florfenicol; CIP, ciprofloxacin; TET, tetracycline; MEM, meropenemS/T; TIG, tigecycline; trimethoprim/sulfamethoxazole). [file Image_2.TIF]
